# Primary Sarcomatoid Malignant Mesothelioma of the Pancreas

**DOI:** 10.1007/s11605-018-3994-4

**Published:** 2018-10-08

**Authors:** Yajie Zhao, Zhuo Li, Chengfeng Wang

**Affiliations:** 1 Department of Pancreatic and Gastric Surgery, National Cancer Center/National Clinical Research Center for Cancer/Cancer Hospital, Chinese Academy of Medical Sciences and Peking Union Medical College, Beijing, 100021 China; 2 Department of Pathology, National Cancer Center/National Clinical Research Center for Cancer/Cancer Hospital, Chinese Academy of Medical Sciences and Peking Union Medical College, Beijing, 100021 China

**Keywords:** Sarcomatous mesothelioma, Pancreatic tumor

## Case Presentation

**Fig. 1:**
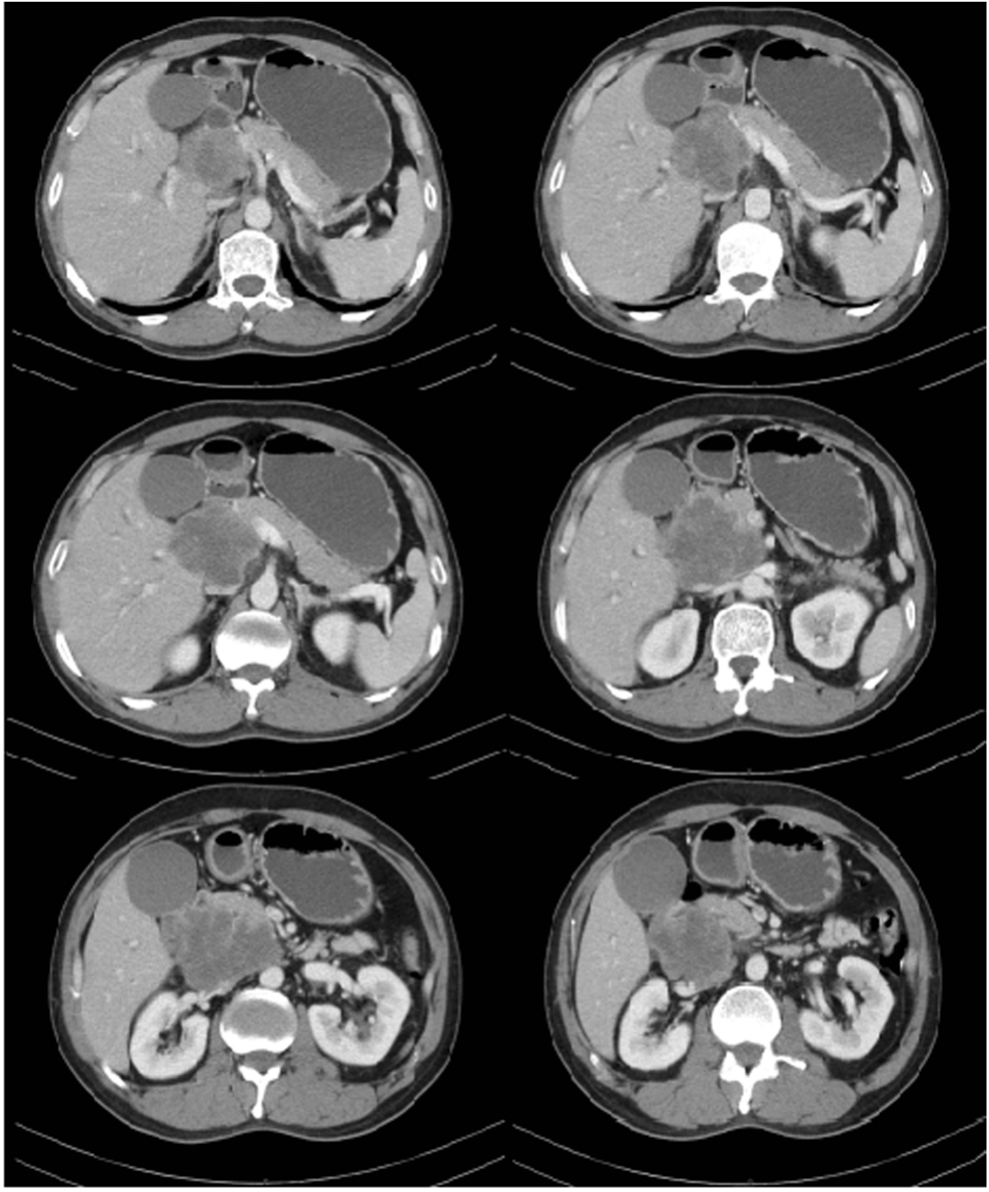
CT: The enhancement of tumor is inhomogeneous, has fuzzy boundaries, and involved the common bile duct, duodenum. The tumor was adjacent to the portal vein, the superior mesenteric vein, the inferior vena cava, and the right renal vein

**Fig. 2:**
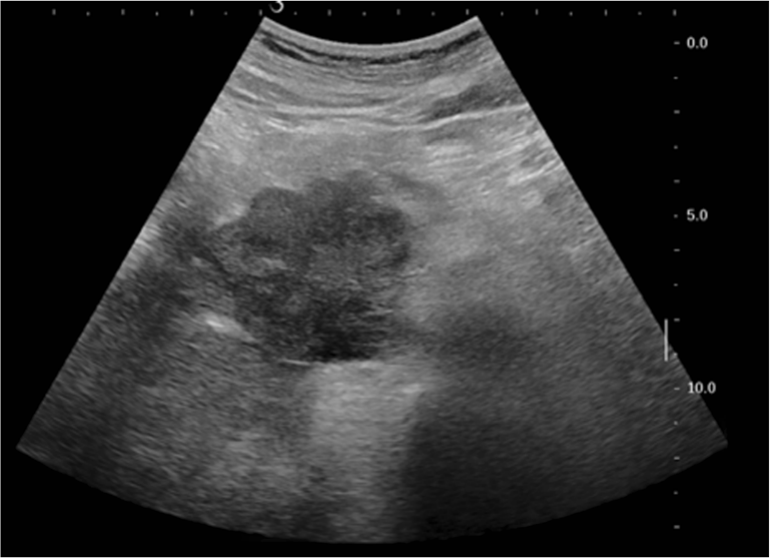
Ultrasound: An irregular, lobulated mass in the head of the pancreas

**Fig. 3:**
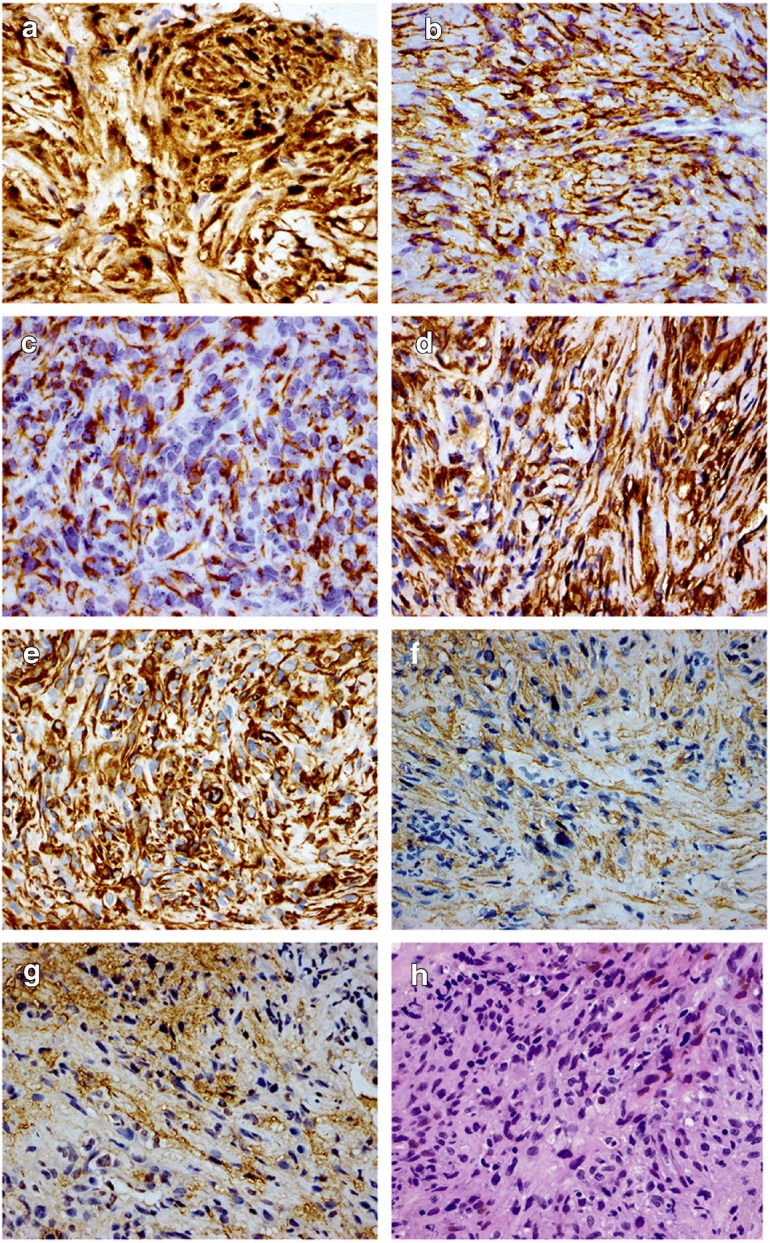
Immunohistochemically (× 40) positive for calretinin (+++) (**A**)**,** D2-40 (++) (**B**), AE1/AE3 (++) (**C**)**,** CK18 (+++) (**D**), Vim (++) (**E**), SMA (+) (**F**)**,** MC (+) (**G**)**,** and tumor consisted of spindle cells and numerous mitotic figures were evident (HE, × 20) (**G**)

A 51-year-old male presented with yellow discoloration of skin, previously healthy, back pain, and weight loss for 1 month. He had no history of asbestos exposure. Laboratory tests of liver function revealed [alanine transaminase (ALT), 244 U/L (range 9–50); aspartate transaminase (AST), 159 U/L (range 15–40); alkaline phosphatase (ALP), 637 U/L (range 45–125); total bilirubin (TBIL), 139.9 μmol/L(range 1.71–17.1); direct bilirubin (DBIL), 119.4 μmol/L (range 0–5.1)]. The patient’s serum carcinoembryonic antigen (CEA) level and CA19-9 level was normal. No abnormality was observed on chest X-ray examination. Abdominal post-contrast CT scan revealed an irregular, lobulated mass (maximum cross-section, 7.8 × 6.8 cm) in the head of the pancreas. The tumor enhancement was heterogeneous with indistinct boundaries, and the common bile duct and duodenum were involved. The tumor was adjacent to the portal vein, the superior mesenteric vein, the inferior vena cava, and the right renal vein. No enlarged lymph nodes were observed in the pelvic cavity, retroperitoneum, or bilateral inguinal region (Fig. 1). An ultrasound-guided fine-needle aspiration biopsy of the pancreatic tumor was performed (Fig. 2). Histologically, the tumor consisted of spindle cells, and numerous mitotic figures were evident. Immunohistochemical staining revealed that the tumor cells were positive for calretinin, D2-40, AE1/AE3, CK18, Vim, SMA, and MC; however, they were negative for CD34, CD117, CK5/6, CK7, CEA, DOG1, desmin, S100, WT1, and Ki-67 (20%+) (Fig. 3). The findings were consistent with a diagnosis of primary intra-pancreatic sarcomatoid malignant mesothelioma (SMM).

The tumor was not amenable to radical surgery because of its location and size. The patient was treated with palliative chemotherapy. After two cycles of pemetrexed plus cisplatin, efficacy evaluation revealed progressive disease. The patient decided to cease chemotherapy and received the best supportive care. The patient died of cachexia 4 months post-diagnosis.

## Discussion

Primary malignant mesotheliomas rarely develop in solid organs, and little is known about the behavior of such tumors. Here, we provide the first report of a case of intra-pancreatic SMM. Clinical diagnosis of pancreatic mesothelioma is difficult because of a lack of specific clinical symptoms and CT/MRI imaging findings. Histopathological and immunohistochemical examinations play an important role in the diagnosis of mesothelioma. Unlike conventional peritoneal mesothelioma, peritoneum metastasis and cavity effusion are rare at the time of initial diagnosis in solid organ cases, such as the liver^1^ and the spleen.^2^ The therapy for pancreatic mesothelioma is primarily surgical. Radiotherapy and chemotherapy have very limited effect in pancreatic malignant mesothelioma, and non-surgical therapy has only been suggested in a few cases with very extensive and inoperable lesions. The prognosis of pancreatic mesothelioma is different depending on the pathological type and stage. Rosanny et al.^3^ reported a case of intra-pancreatic epithelioid malignant mesothelioma. Follow-up revealed no evidence of residual or recurrent disease 32 months after pancreatoduodenectomy without adjuvant radiotherapy and chemotherapy. However, in our reported case, the tumor was not amenable to radical surgery because of its late stage. Although the patient was treated using palliative chemotherapy, the patient did not benefit as an efficacy evaluation revealed progressive disease after two treatment cycles, and the survival period was approximately 4 months after diagnosis. The prognosis of intra-pancreatic SMM may be poor because of its highly invasive behavior, local recurrence, and metastasis.
